# On the Effects of Diluted and Mixed Ionic Liquids as Liquid Substrates for the Sputter Synthesis of Nanoparticles

**DOI:** 10.3390/nano10030525

**Published:** 2020-03-14

**Authors:** Michael Meischein, Marvin Fork, Alfred Ludwig

**Affiliations:** Materials Discovery and Interfaces, Institute for Materials, Faculty of Mechanical Engineering, Ruhr University Bochum, Universitätsstr. 150, D-44780 Bochum, Germany; michael.meischein@rub.de (M.M.); marvin.fork@rub.de (M.F.)

**Keywords:** ionic liquids, sputter deposition, nanoparticles, ionic liquid mixtures

## Abstract

The synthesis of nanoparticles by combinatorial sputtering in ionic liquids is a versatile approach for discovering new materials. Whereas the influence on nanoparticle formation of different pure ionic liquids has been addressed, the influence of (I) dilution of ionic liquid with solvents and (II) different mixtures of ionic liquids is less known. Therefore, mixtures of the ionic liquid [Bmim][(Tf)_2_N] with the organic solvent anisole and other ionic liquids ([Bmim][(Pf)_2_N], [BmPyr][(Tf)_2_N]) were used as liquid substrates for the sputter synthesis of nanoparticles, in order to investigate the influence of these mixtures on the size of the nanoparticles. First, mixtures of anisole with a suspension of sputtered Ag nanoparticles in [Bmim][(Tf)_2_N] were prepared in different volumetric steps to investigate if the stabilization of the NPs by the ionic liquid could be reduced by the solvent. However, a continuous reduction in nanoparticle size and amount with increasing anisole volume was observed. Second, Ag, Au and Cu were sputtered on ionic liquid mixtures. Ag nanoparticles in [Bmim][(Tf)_2_N]/[Bmim][(Pf)_2_N] mixtures showed a decrease in size with the increasing volumetric fraction of [Bmim][(Tf)_2_N], whereas all nanoparticles obtained from [Bmim][(Tf)_2_N]/[BmPyr][(Tf)_2_N] mixtures showed increasing size and broadening of the size distribution. Maximum sizes of sputtered Ag and Au NPs were reached in mixtures of [Bmim][(Tf)_2_N] with 20 vol.% and 40 vol.% [BmPyr][(Tf)_2_N]. The results indicate that ionic liquid mixtures with different portions of cations and anions have the capability of influencing the ionic liquid stabilization characteristics with respect to, e.g., nanoparticle size and size distribution.

## 1. Introduction

Ambits of metal nanoparticles (NPs), like catalysis [1,2,3,4], medical applications [5,6,7,8] and as additives in electrolytes [9,10,11], show their technological and scientific significance. For enabling applications of sputtered NPs in ionic liquids (ILs), studies of their stabilization procedure and possible improvements thereof, including tuning capabilities concerning their formation, are necessary. The most common technique for NP synthesis in a liquid environment is the wet chemical approach using metal precursors which are decomposed or reduced by chemical reactions [12,13,14,15,16]. However, this approach can have the drawback of byproducts remaining in the final NP solution from the mentioned reactions which can affect the NP size [17] and the general applicability of the NPs [18,19]. The physical vapor deposition of metals in ionic liquids (ILs) [18,20,21,22,23,24,25] where ILs [26,27] serve as a liquid “substrate” can avoid these drawbacks. ILs are liquid salts consisting of appropriate cations and anions with melting points at temperatures <100 °C. Many ILs are liquid at room temperature [28]. Due to the high number of suitable cations and anions, for enabling the theoretical fabrication of 10^18^ ILs [29], a task-specific IL can be designed for nearly every desired application. Since ILs have a negligible vapor pressure [27,30,31], they are frequently used as substrate or solvents for ultrahigh vacuum and green chemistry processes [32]. Additionally, the outstanding chemical and physical characteristics of ILs make them ideal for applications in catalysis [33] and as scavenging and reaction media for industry [34].

One physical vapor deposition synthesis route is the thermal evaporation of a metal on the surface of an IL [20,21,22]. However, the synthesis of binary or higher order NPs with this method is complicated. Since each element has its specific evaporation temperature and vapor pressure, an adjustment of a desired alloy composition is challenging [24]. The sputter deposition of metals in ILs [18,23,24,25] is an alternative physical vapor deposition synthesis pathway, permitting the fabrication of custom-designed multinary NPs. These can be synthesized with adjustable multinary composition and crystal structure, depending on the used sputter technique [35]. The chosen IL substrate for the sputter deposition controls the size and morphology of the NPs, but without the harm of (organic) impurities or byproducts [18,19]. One advantage of sputter and co-sputter depositions is that multinary systems in form of forced solid solutions can be fabricated, even when the elements are immiscible in bulk [36]. The tunable multinary NP composition, which enables the synthesis of tailored NPs for specific applications, is controlled by the deposition rates of the specific sputter sources [24,37], also influencing the NP growth process [38,39]. Additionally, sputter deposition is not limited by the availability of precursor chemicals or their reaction pathway, since, in principle, all vacuum compatible elements can be combined into multinary NPs by co-sputtering [23,24,25].

Sizes and size distributions of the NPs, which are further important characteristics for the fabrication of task-designed NPs, are mainly controlled by the used IL, as the structure of the IL ions controls the NP stabilization. Since the NP surface is known to have a lack of electrons [40,41], the negatively charged anions of an IL electrostatically form a first layer around the NP surface, which is then surrounded by the opposite charged cation of the IL. Thus, a protective double layer of IL ions around the NPs is developed [42,43]. The same charge of the surrounding protective layers leads to an electrostatic repulsion between the surrounded NPs, preventing agglomeration. Additionally, a steric stabilization occurs: Its strength depends on the coverage of the NPs with cations, having an alky chain dangling away from the NP surface [42,43]. The alkyl chains act as a protective cage, preventing the physical contact of the NPs by sort of pushing approaching NP cages away and impeding chemical bonds. The caged NPs are thus incorporated by a semi-organized nanostructure existing around the electric double layer cage into the IL network. This network is formed by electrostatic interactions and hydrogen bonds between the single IL ions [44], making the NPs movable within the IL but prevented from agglomeration [42]. The NP size depends on several structural and electrostatic characteristics of the IL ions being responsible for the strength of the NP cage and the general formation process during sputter deposition. Several publications report that an increasing alkyl chain length of the cations is directly linked to a decreasing NP diameter and size distribution, since longer alkyl chains increase the organization range of the specific IL and thus better prevent the caged NP from making physical contact [19,42,45]. Additionally, increasing the volume of the used IL anion is correlated with an increasing of the NP diameters stabilized in the specific ILs [46,47]. This effect occurs because the charge distributed over the anion stays constant (one elemental charge) and, thus, the charge density distributed over the anion decreases if the anion volume increases. Therefore, a weakening of the first stabilization shell of the NP cage due to reduced electrostatic forces occurs. Because of this reduction in the charge density in the case of increasing anion volumes, the IL organization and stabilization ability is reduced [48].

These stabilization factors mainly work inside the IL “bulk”. However, for the general nucleation and growth process of sputtered NPs on IL, the first interactions with the IL surface are considered to be the most important for the NP size [19,49]. Factors like surface structure, surface composition and ion orientation at the surface, macroscopically compiled into IL surface tension and viscosity [49], do also depend on the specific IL ion structures and interactions [50]. For example, it is assumed that the surface tension of the IL is directly correlated to the time the NPs spend on the IL surface and thus can grow due to further incoming atoms before being immersed into the IL “bulk” [19,49]. The viscosity is assumed to affect the subsequent behavior of the NPs within the IL, concerning possible aggregation and agglomeration due to the specific diffusion velocity [19].

By applying this information about stabilization factors and growth processes for the choice of the specific IL, the targeted NP size and size distribution can be reliably controlled. However, each IL seems to have a limit for stabilized NP sizes [23]. The size of sputtered NPs in ILs is typically <10 nm [23,24,25,51,52].

**Figure 1:**
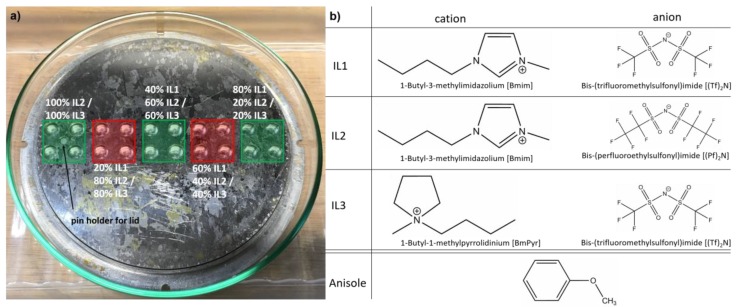
(**a**) Photo of the 20-cavity plate (100 mm diameter). The applied arrangement of ionic liquid (IL) (mixtures) is indicated. **IL1** was mixed with **IL2** or **IL3** respectively in steps of 20 vol.% from pure **IL2/IL3** to a mixture of 20 vol.% **IL2/IL3** with 80 vol.% **IL1** before deposition. Anisole was added in the same volumetric steps to already-sputtered pure Ag in **IL1**. The chemical structures of the IL ions and anisole are shown in (**b**). **IL2** and **IL3** differ only by the anion and cation, respectively, with respect to **IL1**. The drawings of the chemical structures are not depicted in the same size format (drawn with “ChemDraw Professional” from PerkinElmer).

For some applications and for the purpose of in-depth studies it would be desirable to be able to achieve NP sizes up to several tens of nanometers. Here, we investigate if it is possible to destabilize the NP cage in a controlled way so that the NPs can grow bigger than the actual stabilization limit of a pure IL would allow. To this end, we destabilized the IL 1-butyl-3-methylimidazolium bis-(trifluoromethylsulfonyl)imide [Bmim][(Tf)_2_N] with anisole as an organic solvent and with two other ILs. Anisole, as a diluting solvent, was chosen since it is well solvable in ILs and does not destroy the ILs [53]. The used ILs 1-butyl-3-methylimidazolium bis-(perfluoroethylsulfonyl)imide [Bmim][(Pf)_2_N] and 1-butyl-1-methylpyrrolidinium bis-(trifluoromethylsulfonyl)imide [BmPyr][(Tf)_2_N] have been chosen so that only one ion, for [Bmim][(Pf)_2_N] the anion and for [BmPyr][(Tf)_2_N] the cation, differs from [Bmim][(Tf)_2_N], to investigate only the effects caused by one structural change, as depicted in Figure 1.

IL mixtures have been applied in the past for testing their general characteristics [54,55,56,57] as improved electrolyte for solar cells [58] and as stabilization matrices for NPs [59,60] and carbon nanotubes [61]. IL mixtures showed improved characteristics, like increased conductivity [54], gas solubility [56] and power conversion efficiencies [58] with respect to pure ILs. However, the application of IL mixtures as substrates for NP synthesis by sputter deposition to influence the NP size distribution has not been conducted so far. Our sputter setup allows simultaneous sputtering in four different binary IL mixtures in one experiment. Thus, identical deposition parameters are achieved for each mixture, and the obtained results can be directly correlated to the specific volumetric IL mixtures. The success of our approach of sputtering on clean, unused IL mixtures as a new method for targeted NP size control and for understanding the general NP formation processes in the ILs is confirmed by our results. Thus, we want to emphasize the suitability of sputtering on IL mixtures as new synthesis method for targeted NP size tuning.

## 2. Materials and Methods 

The description of the synthesis routes is divided into sputter-deposition in IL mixtures and the dilution of the already-sputtered Ag in IL with anisole, since the mixture procedure was different. The TEM grid preparation was identical for all samples. ILs were purchased from Iolitec (Heilbronn, Germany) and stored and processed under Ar atmosphere in a glovebox with water and oxygen contents <0.5 ppm; no further purification was performed. Anisole was purchased from Acros Organics (Geel, Belgium). The characteristics of the chemicals are listed in Table 1. For simplification, the IL names are abbreviated as **IL1** for [Bmim][(Tf)_2_N], **IL2** for [Bmim][(Pf)_2_N] and **IL3** for [BmPyr][(Tf)_2_N] (see Figure 1).

### 2.1. Depositions of Ag, Cu, Au into IL and IL Mixtures

Sputter depositions were performed in a commercial co-sputter system (AJA POLARIS-5 from *AJA* INTERNATIONAL, Inc., North Scituate, MA, USA) with 1.5-inch diameter magnetron sputter cathodes and multiple sputter source DC power supplies (DC-XS 1500 from *AJA* INTERNATIONAL, Inc., North Scituate, MA, USA). Ar was used as processing gas (purity 99.999%, Praxair, Düsseldorf, Germany). An Ag target (Sindlhauser Materials, Kempten, Germany), an Au target (Sindlhauser Materials, Kempten, Germany) and a Cu target (EvoChem, Offenbach am Main, Germany), all with the dimensions 38.1 mm diameter × 4.775 mm thick and purity 99.99%, were used. A custom-made cavity plate with an adaptable lid for covering unfilled cavities (see Figure 1) was used to expose the IL mixtures to the sputter flux (for details, see Meyer et al. [23]). The plate and lid were cleaned in an ultrasonic bath (30 min in technical acetone (purity ≥99.5%) and isopropanol (purity ≥99.7%), respectively) and dried in an oven at 80 °C for 60 min. An amount of 35 μL IL (mixture) was filled in each used cavity for all depositions in the glovebox. Pure **IL2** and **IL3** were filled in a square array of four cavities located around the pin holder for the lid (see Figure 1). Since the “dilution” of **IL2** and **IL3** with the “diluting” **IL1** was performed in 20 vol.% steps, which corresponds to 7 μL for 35 μL total volume per cavity, the next array of four cavities was filled with 28 μL **IL2/IL3** and 7 μL **IL1**. This procedure was continued for the next three steps until a mixture of 7 μL **IL2/IL3** and 28 μL **IL1** was present in each of the four cavities of the last cavity array group (see Figure 1). All IL mixing and IL loading procedures into the cavity plate were conducted at room temperature (i.e., around 22 °C) in a glovebox. For analysis of pure **IL2** and **IL3** characteristics for Ag NPs, the TEM data from our previous publication of Ag sputtered in nine different ILs were used [23]. For the analysis of the pure **IL1** characteristics for Au and Cu NPs, the TEM data of a previous publication concerning binary Au-Cu NPs synthesized from unary Au and Cu NPs in IL were used [25].

Before all depositions, the specific IL (mixture) was evacuated in the sputter chamber for at least 72 h to remove remaining water and oxygen, which could be present in the IL or could originate from the transport out of the glovebox positioned opposite the sputter chamber into the chamber, which takes around 10 s. Although no active mixing of the IL mixtures within the cavities was applied, the long evacuation time and the liquid movement due to Brownian motion and water and oxygen bubbles during outgassing are considered to contribute to a homogeneous mixture of the mixed ILs. After plasma ignition (see Table 2 for parameters), the targets were pre-cleaned for 120 s with a closed shutter in front of the cathodes, a plate rotation of 30 rotations per min and a continuous reduction in the Ar pressure to the deposition pressure. The pre-cleaning process removes possible oxide layers from the target surface [23,25]. Since Cu is the least noble of the chosen elements, the specific pre-cleaning time was doubled. After finishing pre-cleaning, the power was set to the specific deposition value and the shutter in front of the cathode was opened for the desired deposition time. The element-specific deposition rates led to different nominal film thicknesses (see Table 2). A film thickness of around 500 nm was intended to achieve sufficient NPs in the IL. The slightly lower film thickness of Au compared to the other elements is related to the price of the Au target and is justifiable with respect to the comparability of the results of the other depositions since the deposition time and thus the film thickness is known to only affect the NP concentration, not the NP size [18,49]. The cathode tilt was adjusted before opening the shutters to an angle of 12° between the normal of the cavity holder and the target normal. Rotation and cathode tilt lead to a homogenous deposition on the substrate. After each deposition, the plate with IL was transferred immediately into Ar atmosphere in the glovebox and the IL was collected and stored there. Whereas the pure ILs are colorless, the NP/IL suspensions showed colors: Au NPs generated a black color with a ruby-colored undertone, whereas Cu NPs caused a black-brown IL color. Ag NPs generated a yellow color in **IL1** and **IL2** and an orange color in **IL3**. No clear color development from yellow to orange was visible in **IL1/IL3**-mixtures for Ag NPs. All mixtures remained mainly yellow.

### 2.2. Dilution of Already-Sputtered Ag in **IL1** with an Organic Solvent

Ag was sputtered in **IL1** as initial state for the experiment since anisole would evaporate before the required sputter pressure in the chamber was reached. Thus, sputter deposition in an IL-anisole mixture is impossible. To overcome this obstacle, Ag NP/**IL1** suspension was filled under Ar atmosphere and at room temperature into Schlenk flasks for further processing. The desired dilution steps are identical to the IL dilution steps but with a bigger total volume of 100 μL: i.e., four Schlenk flasks were prepared with Ag IL volumes from 80 μL to 20 μL in 20 μL steps and equipped with a magnetic stir bar. The flasks were closed under Ar atmosphere for transport out of the glovebox and connected to a Schlenk line. The specific anisole volume to reach 100 μL total liquid volume was filled into the Schlenk flasks under an Ar counter stream via the Schlenk line. After the addition of anisole, the flasks were closed again, and the mixture was stirred for 60 min at 180 rotations per minute in Ar. After mixing, the closed Schlenk flasks were sluiced into the glovebox for storage.

### 2.3. TEM Sample Preparation and Analysis

TEM samples were prepared by dropping 2.5 μL IL (mixture) on the carbon-coated side of holey carbon-coated Au grids (200 mesh, Plano GmbH, Wetzlar, Germany) and leaving the IL at this side for adhesion of NPs for 2.5 h. In order to prevent grid contamination, which could originate from the interaction of the electron beam with IL during TEM, the grids were washed dropwise with dried acetonitrile for 1 h in inert conditions (see supporting information of Meyer et al. [23]) and then stored in Ar atmosphere. Grids were prepared for pure **IL1** samples and all mixtures. For analysis of Ag in pure **IL2** and **IL3**, the data of our previous study of Ag in nine different ILs [23] and, for Au and Cu in pure **IL1**, the data of the previous study concerning binary Au-Cu NPs in ILs [25], were re-analyzed for NP counts. In the previous studies, the grids were turned upside down before washing with acetonitrile. Tests with other samples revealed that the difference in mean NP diameter between turned and unturned grid is 0.1 nm for the value itself as well as the error. Thus, we consider this slight difference in sample preparation as negligible. Conventional TEM and high-resolution TEM (HRTEM) studies were performed using a FEI Tecnai F20 S/TEM instrument operated at 200 kV. For the mean diameter calculation and the size distribution determination, at least 240 NPs per sample were counted.

## 3. Results

### 3.1. Mixture of Ag/**IL1** Suspension with Anisole

**Figure 2:**
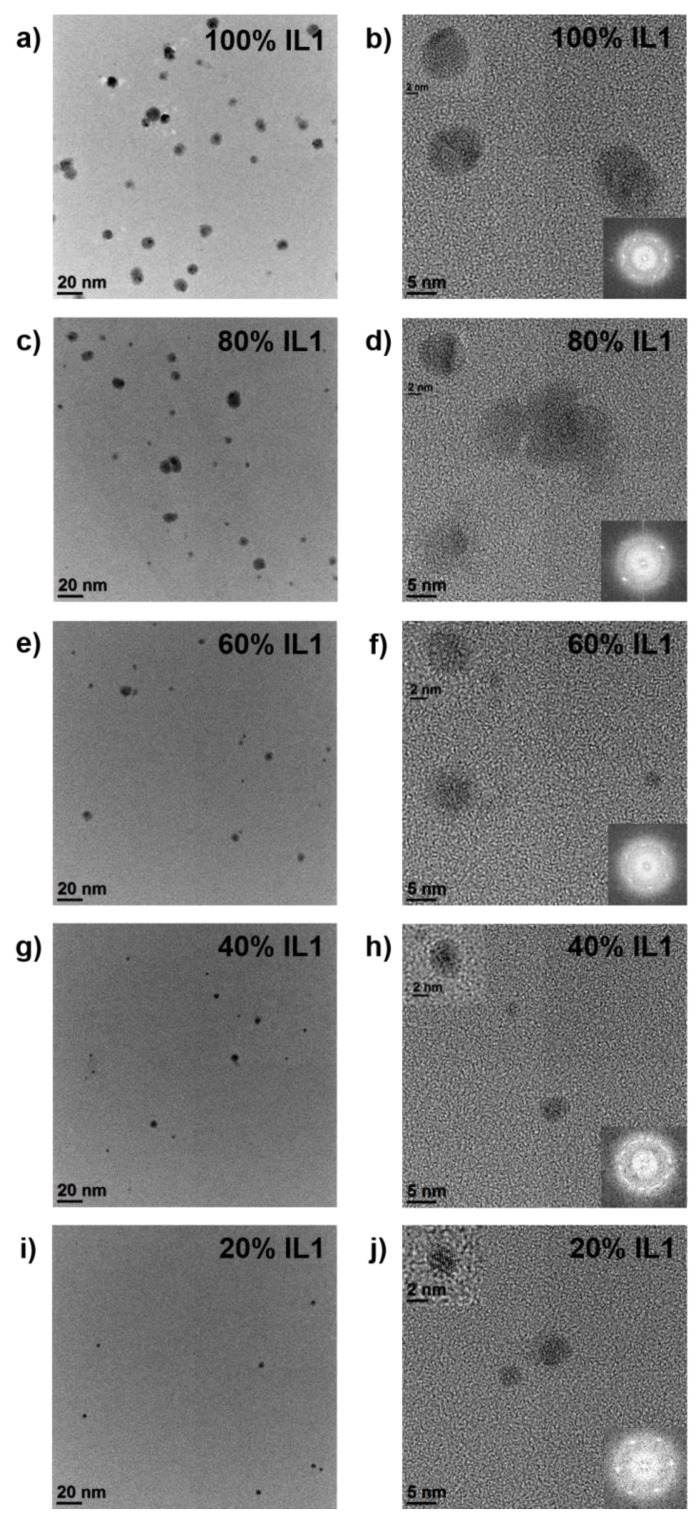
Overview of TEM results of Ag NPs in **IL1** diluted with anisole in 20 vol.% steps. Ag NPs in pure **IL1** are shown in (**a**,**b**). Ag NPs in IL/anisole mixtures of 80 vol.% **IL1** and 20 vol.% anisole are shown in (**c**,**d**), 60 vol.% **IL1** and 40 vol.% anisole in (**e**,**f**), 40 vol.% **IL1** and 60 vol.% anisole in (**g**,**h**), and 20 vol.% **IL1** and 80 vol.% anisole are shown in (**i**,**j**). Fast Fourier Transformation (FFT) and corresponding high-resolution TEM (HRTEM) images of the NPs are displayed as insets in the small overview images.

The NP size and size distribution in the **IL1**/anisole mixtures were analyzed by TEM, see Figure 2. The size of Ag NPs in pure **IL1** was (5.4±2.1) nm. The crystallinity of these NPs was confirmed by lattice fringes visible in HRTEM images, providing also information of a face centered cubic (fcc) structure by Fast Fourier Transformation (FFT) analysis of the image data. Since the amorphous carbon support of the TEM grid also contributes with diffuse rings with high intensities in the pattern to electron diffraction measurements, the determination of the NP crystal structure via diffraction in TEM is difficult. With increasing anisole volume, a significant reduction in NP amount and NP size was observed. Additionally, a constant decrease in the number of crystalline NPs was detected. Due to this substantial reduction in NP amount and size (see Figure 2), no further NP counting was performed.

### 3.2. Ag NPs Sputtered in **IL1/IL2**-Mixtures

**Figure 3:**
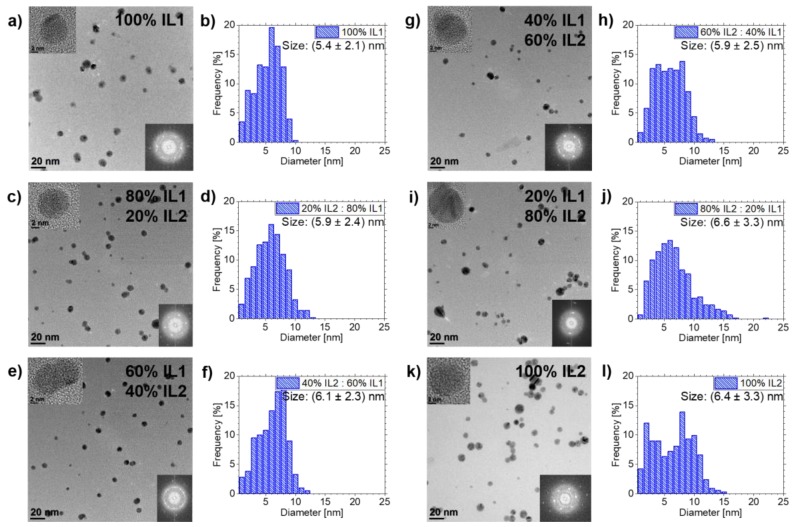
Overview of TEM results of Ag NPs sputtered in **IL1/IL2**-mixtures and pure ILs as references. TEM images and the corresponding NP size distribution are depicted in (**a**,**b**) for **IL1**. Overview images and NP size distributions for the **IL1/IL2**-mixtures are shown in (**c**,**d**) for 80 vol.% **IL1**/20 vol.% **IL2**, in (**e**,**f**) for 60 vol.% **IL1**/40 vol.% **IL2**, in (**g**,**h**) for 40 vol.% **IL1**/60 vol.% **IL2** and in (**i**,**j**) for 20 vol.% **IL1**/80 vol.% **IL2**. Results of pure **IL2** are illustrated in (**k**,**l**). HRTEM images of NPs from the investigated IL (mixtures) and corresponding FFT images are depicted as insets in the overview images, confirming the NP crystallinity due to visible lattice fringes and the corresponding spots in the FFTs.

Using the above-described NP size analysis procedure, Ag NPs sputtered in **IL1**/**IL2**-mixtures were investigated. NP stabilization was successful in pure ILs and in all mixtures, see Figure 3. The Ag NPs showed fcc structure, as determined by analyzing FFT data of HRTEM images.

The mean sizes of the Ag NPs with increasing volumetric portion of **IL2** are as follows: Starting with (5.4±2.1) nm for pure **IL1**, the NP diameter increases to (5.9±2.4) nm for 80 vol.% **IL1**/20 vol.% **IL2**, (6.1±2.3) nm for 60 vol.% **IL1**/40 vol.% **IL2**, (5.9±2.5) nm for 40 vol.% **IL1**/60 vol.% **IL2**, (6.6±3.3) nm for 20 vol.% **IL1**/80 vol.% **IL2** and (6.4±3.3) nm for pure **IL2**. Additionally, the size distribution broadens with increasing **IL2** fraction towards bigger NPs with respect to pure **IL1**. The biggest NP for pure **IL1** has a diameter of 10 nm, whereas the biggest NP for pure **IL2** has a diameter of 15 nm. The biggest NPs (22 nm diameter) in this mixture were detected for 20 vol.% **IL1**/80 vol.% **IL2**.

### 3.3. Ag NPs Sputtered in **IL1/IL3**-Mixtures

**Figure 4:**
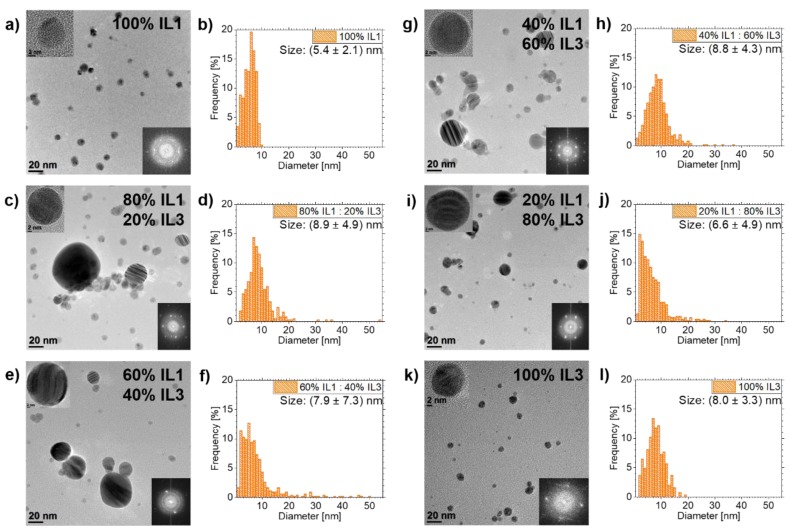
Overview of TEM results of Ag NPs sputtered in **IL1/IL3**-mixtures and pure ILs as references. TEM images and the corresponding NP size distribution are depicted in (**a**,**b**) for pure **IL1**. Overview images and NP size distributions for the **IL1/IL3**-mixtures are shown in (**c**,**d**) for 80 vol.% **IL1**/20 vol.% **IL3**, in (**e**,**f**) for 60 vol.% **IL1**/40 vol.% **IL3**, in (**g**,**h**) for 40 vol.% **IL1**/60 vol.% **IL3** and in (**i**,**j**) for 20 vol.% **IL1**/80 vol.% **IL3**. Results of pure **IL3** are illustrated in (**k**,**l**). HRTEM images of NPs from the particular investigated IL (mixtures) and corresponding FFT images are depicted as insets in the overview images, confirming the NP crystallinity due to the visible lattice fringes and the corresponding spots in the FFTs.

All mixtures showed stable NPs, but the stabilization effect was different compared to **IL1**/**IL2**-mixtures, see Figure 4. The fcc structure of the Ag NPs was determined by analyses of FFT data from HRTEM images of all samples in this mixture row. Whereas Ag NPs in **IL1** have a mean diameter of (5.4±2.1) nm, NP diameters in the mixtures change to (8.9±4.9) nm for 80 vol.% **IL1**/20 vol.% **IL3**, (7.9±7.3) nm for 60 vol.% **IL1**/40 vol.% **IL3**, (8.8±4.3) nm for 40 vol.% **IL1**/60 vol.% **IL3**, (6.6±4.9) nm for 20 vol.% **IL1**/80 vol.% **IL3** and (8.0±3.3) nm for pure **IL3**. The NP size distributions are different for mixtures and pure ILs (see Figure 4). For pure ILs, the size distributions are much narrower compared to IL mixtures. Pure **IL1** leads to the smallest maximum NP diameter of 10 nm. For pure **IL3**, the maximum NP diameter of 19 nm is nearly twice the maximum diameter of **IL1**. However, all **IL1/IL3**-mixtures show much bigger maximum NP diameters with respect to pure ILs, starting with 34 nm for 20 vol.% **IL1**/80 vol.% **IL3** ranging up to 54 nm for 80 vol.% **IL1**/20 vol.% **IL3**. The most NPs above 20 nm diameter are present in the mixture 60 vol.% **IL1**/40 vol.% **IL3**. 

### 3.4. Au NPs Sputtered in **IL1/IL3**-Mixtures

**Figure 5:**
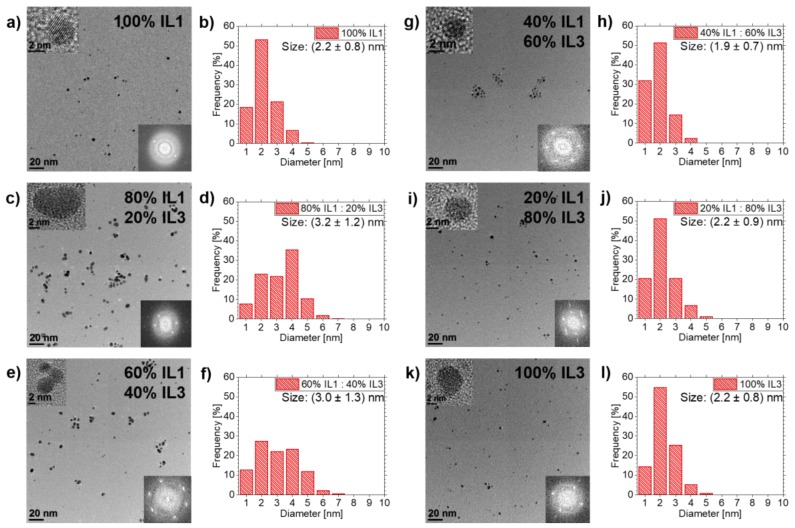
Overview of TEM results of Au NPs sputtered in **IL1/IL3**-mixtures and pure ILs as references. TEM images and the corresponding NP size distribution are depicted in (**a**,**b**) for pure **IL1**. Overview images and NP size distributions for **IL1/IL3**-mixtures are shown in (**c**,**d**) for 80 vol.% **IL1**/20 vol.% **IL3**, in (**e**,**f**) for 60 vol.% **IL1**/40 vol.% **IL3**, in (**g**,**h**) for 40 vol.% **IL1**/60 vol.% **IL3** and in (**i**,**j**) for 20 vol.% **IL1**/80 vol.% **IL3**. Results of pure **IL3** are illustrated in (**k**,**l**). HRTEM images of NPs from the particular investigated IL (mixtures) and corresponding FFT images are depicted as insets in the overview images, confirming NP crystallinity due to visible lattice fringes and corresponding spots in the FFTs.

The findings of Ag NPs sputtered in **IL1**/**IL3**-mixtures were compared with the results of sputtering Au in the same **IL1**/**IL3**-mixtures, as the influence of this IL mixture was more pronounced than in **IL1**/**IL2**-mixtures. Figure 5 shows the results: Au NPs have fcc structures, as determined by analyses of FFT data from HRTEM images of all samples in this mixture row. The Au NP mean diameters show a similar trend to the Ag NPs in **IL1**/**IL3**-mixtures, however with notable differences. **IL1** stabilizes Au NPs with a mean diameter of (2.2±0.8) nm. For IL mixtures, the NP diameters differ: (3.2±1.2) nm for 80 vol.% **IL1**/20 vol.% **IL3**, (3.0±1.3) nm for 60 vol.% **IL1**/40 vol.% **IL3**, (1.9±0.7) nm for 40 vol.% **IL1**/60 vol.% **IL3** and (2.2±0.9) nm for 20 vol.% **IL1**/80 vol.% **IL3**. **IL3** stabilizes Au NPs with a mean diameter of (2.2±0.8) nm. The difference in the size distributions and maximum NP sizes is not as pronounced as for Ag NPs but shows again that the **IL1**/**IL3**-mixtures tend to have a broader size distribution than pure ILs, stated by the mixtures 80 vol.% **IL1**/20 vol.% **IL3** and 60 vol.% **IL1**/40 vol.% **IL3** (see Figure 5). Both mixtures have a bigger mean NP diameter than both pure ILs. The NP frequencies for each diameter are distributed over seven sizes instead of five for the pure ILs. Additionally, those two mixtures lead to the biggest maximum NP size of 7 nm, whereas pure ILs only have 5 nm maximum NP size. For Ag NPs in **IL1**/**IL3**-mixtues, the same behavior concerning maximum NP size was determined for those particular mixtures.

### 3.5. Cu NPs Sputtered in **IL1/IL3**-Mixtures

**Figure 6:**
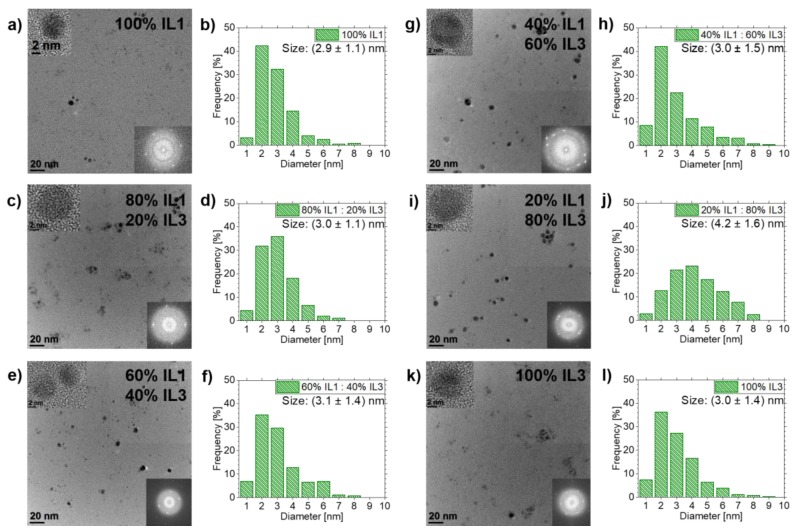
Overview of TEM results of Cu NPs sputtered in **IL1/IL3**-mixtures and pure ILs as references. TEM overview images and the corresponding NP size distribution are depicted in (**a**,**b**) for **IL1**. Overview images and NP size distributions for **IL1/IL3**-mixtures are shown in (**c**,**d**) for 80 vol.% **IL1**/20 vol.% **IL3**, in (**e**,**f**) for 60 vol.% **IL1**/40 vol.% **IL3**, in (**g**,**h**) for 40 vol.% **IL1**/60 vol.% **IL3** and in (**i**,**j**) for 20 vol.% **IL1**/80 vol.% **IL3**. Results of **IL3** are illustrated in (**k**,**l**). HRTEM images of NPs from the investigated IL (mixtures) and corresponding FFT images are depicted as insets in the overview images, confirming the NP crystallinity due to the visible lattice fringes and the corresponding spots in FFTs.

The results of Cu NPs sputtered in **IL1**/**IL3**-mixtures, Figure 6, show a different trend compared to the results for Ag and Au. Cu NPs show as well fcc structure, as determined by analyses of FFT data from HRTEM images. However, the mean NP diameters do not vary much for different IL mixtures. Cu NPs in **IL1** have a mean diameter of (2.9±1.1) nm. NPs in IL mixtures show mean diameters of (3.0±1.1) nm for 80 vol.% **IL1**/20 vol.% **IL3**, (3.1±1.4) nm for 60 vol.% **IL1**/40 vol.% **IL3**, (3.0±1.5) nm for 40 vol.% **IL1**/60 vol.% **IL3** and (4.2±1.6) nm for 20 vol.% **IL1**/80 vol.% **IL3**. The mean diameter of Cu NPs in pure **IL3** is (3.0±1.4) nm. Only the mean diameter of the 20 vol.% **IL1**/80 vol.% **IL3** mixture is more than 1 nm bigger than that of the other samples. Additionally, all samples show basically comparable size distributions except 20 vol.% **IL1**/80 vol.% **IL3**, whose size distribution is shifted to bigger NP diameters (see Figure 6).

## 4. Discussion

### 4.1. NP Stabilization in IL Mixed with Organic Solvent

The aim of this experiment was to weaken the NP stabilization in a controlled manner by adding the organic solvent anisole, in order to enable NP growth above the size limit for a pure IL. Since the reduction in NP size and amount is obvious for increasing anisole content in the mixture, the effect of the anisole concerning NP stabilization in the IL surpasses the aimed controlled weakening of the stabilization, which should result in bigger NPs. The NP stabilization can be described by a protective double layer of IL ions around the NP generating electrostatic repulsion of “caged NPs” and by steric stabilization due to alkyl chains of the IL cation dangling away from the NP surface [42,43]. Anisole is well solvable in ILs due to its methoxy group [53]. Therefore, anisole molecules should be present everywhere in the IL, also in the vicinity around the “caged NPs”, probably in direct contact with the metal surface. Thus, they reduce the IL anion concentration in the first protective layer. This affects the strength of the electrostatic stabilization of the NPs since anisole has no partial charge in contrast to IL ions. Additionally, anisole molecules take the place of cations in the second protective layer and thus reduce the steric stabilization, since they have no long alkyl chain (see Figure 1). Therefore, we assume that the stabilization by the protective cages around the metal NPs is impeded by anisole molecules being present among the IL ions. With increasing anisole content, the IL ions are more and more suppressed by anisole and the stabilization cage becomes weaker with increasing anisole overtaking IL ion places there. Thus, the NP ability to agglomerate and fall out of the IL stabilization matrix increases. Due to this, we assume that the Ag in the IL is more and more present in agglomerations instead of being stabilized in NPs, explaining the continuous reduction in NP amount and size with increasing anisole content.

### 4.2. Effects of IL Mixtures on NPs Sizes

**Figure 7:**
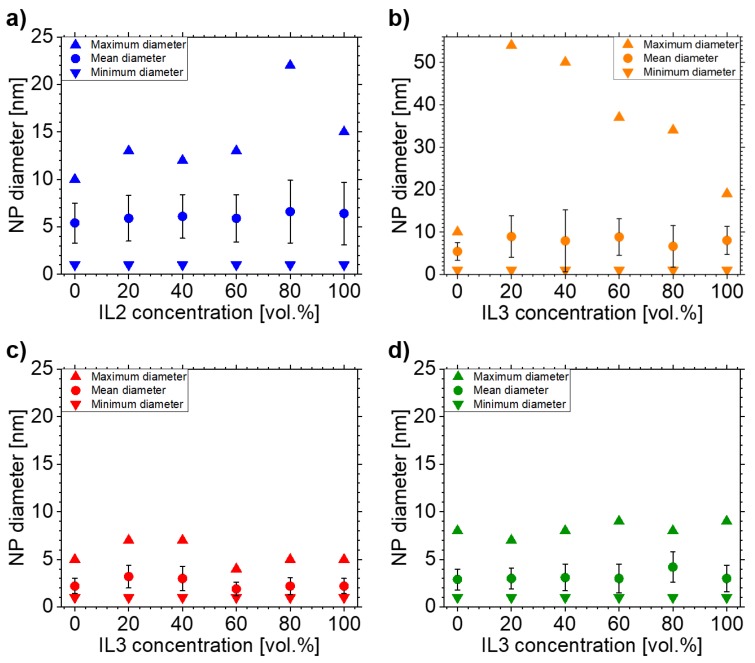
Overview of the different size parameters of all NP/IL-mixture combinations. Minimum and maximum NP diameter as well as the mean diameter of the NPs with error are depicted in (**a**) for Ag in **IL1/IL2**-mixtures, in (**b**) for Ag in **IL1/IL3**-mixtures, in (**c**) for Au in **IL1/IL3**-mixtures and in (**d**) for Cu in **IL1/IL3**-mixtures.

A control of NP sizes was reached by mixtures of **IL1** with **IL2** and **IL3**, in contrast to the destabilization of **IL1** with anisole. The size characteristics for each NP/IL-mixture combination reported above are comprehensively visualized in Figure 7. As stated in the introduction, the effects concerning NP diameters differ between the different IL mixtures. The results shown in Figure 7 are discussed below.

### 4.3. NP Stabilization in **IL1/IL2**-Mixtures

NPs were stabilized in **IL1/IL2**-mixtures, with the NP size depending on the IL mixture. Both ILs have the same cation [Bmim] but differ in the anion, which is [(Tf)_2_N] for **IL1** and [(Pf)_2_N] for **IL2**. We assume that the differences in NP sizes for the **IL1/IL2**-mixtures only depend on the differences in the anion sizes of the used ILs. Although the NP nucleation process is expected to occur mainly at the IL surface [19,49], the surface conditions for **IL1** and **IL2** should be comparable, since the anions are expected to be mainly present in the “bulk” IL phase, leaving the surface population to the cation (if it has a comparably long alkyl chain) [49]. For **IL1** and **IL2** (both [Bmim]), the alkyl chain length is identical. Thus, the process responsible for the NP size differences is assumed to also take place in the IL “bulk” after the previous NP formation on the IL surface. As depicted in Figure 1 and noted in Table 1, the [(Pf)_2_N] anion is bigger concerning volume than [(Tf)_2_N], but both have a comparable structure. Both ions have the same charge, but in the case of [(Pf)_2_N] the charge is distributed over a volume approximately 25% bigger than the volume of [(Tf)_2_N], decreasing the interaction energy and cation-anion cohesion energy, as reported by Fernandes et al. for increasing anion radii [50]. Thus, the charge density of the [(Pf)_2_N] anion is lower compared to [(Tf)_2_N]. Additionally, due to the bigger [(Pf)_2_N] volume, the number of [(Pf)_2_N] anions in the first stabilizing layer of the NP cage is less compared to [(Tf)_2_N] for the lack of space. Both characteristics of the [(Pf)_2_N] lead to a lower coordinative ability and thus a weaker NP stabilization with respect to [(Tf)_2_N]. This means that, with increasing **IL2** and thus increasing [(Pf)_2_N] portion in the IL mixture, the NP size should also increase due to more likely physical interactions between the caged NPs which then can grow together. Figure 3 and Figure 7a indicate that a linear increase in the mean NP size and broadening of the size distribution towards bigger NPs occurs with increasing **IL2** portion, which supports these assumptions. Additionally, several other studies found an identical behavior of stabilized NPs, however they did not compare IL mixtures of different compositions, but pure ILs with the same cations but different anions and thus different anion volumes [46,47]. In these cases, the NP sizes differ by several nm up to tens of nm. In contrast, our approach shows only a slight size increase of 1.2 nm (the biggest increase). Thus, our approach gives the opportunity for a much finer tuning of the desired NP sizes by adjusting the appropriate IL mixture and confirms the dependency of NP size on anion volume. The maximum NP diameter is not as strongly influenced by the **IL1/IL2**-mixture compared to the **IL1/IL3**-mixture. All **IL1/IL2**-mixtures show comparable maximum NP diameters with respect to **IL2**, except the mixture 20 vol.% **IL1**/80 vol.% **IL2**, where only one NP had a large size. However, this single incident is not statistically significant.

### 4.4. NP Stabilization in **IL1/IL3**-Mixtures

NPs could be stabilized with controlled sizes in **IL1/IL3**-mixtures. However, the results are different to the **IL1/IL2**-mixtures, as depicted in Figure 4 and Figure 7b for Ag NPs. The ILs of the **IL1/IL3**-mixture have the same anion [(Tf)_2_N] but differ in the cation ([Bmim] for **IL1**, [BmPyr] for **IL3**). By investigating these mixtures, the effect of the cation on NP formation and stabilization was revealed. The cations have slightly different volumes, with [BmPyr] being bigger than [Bmim] (see Table 1) and both show a different structure (see Figure 1). [Bmim] and [BmPyr] have comparable alkyl chain lengths, but the central ring structures show two disparities. For [BmPyr], there is only one nitrogen atom in the pyrrolidinium ring, whereas [Bmim] has two nitrogen atoms in the imidazolium ring. Additionally, [BmPyr] has no covalent double bonds in the ring, whereas [Bmim] has two and thus an aromatic character. The delocalization of charges in aromatic rings (like the imidazolium ring) leads to weaker electrostatic strengths with respect to fixed localized charges in saturated rings (e.g., the pyrrolidinium ring) and thus ILs based on imidazolium rings with aromatic character have a lower relative interaction strength than ILs with saturated rings [50]. Since it is expected that the NP formation predominantly occurs on the IL surface [19,49] and anions tend to be more present in the IL “bulk” if the cation has at least a butyl chain [19], the cations dominate the surfaces of **IL1/IL3**-mixtures. Since the [BmPyr] cation of **IL3** has no aromatic character in contrast to [Bmim] of **IL1**, it is expected that the organization strength of the cation-dominated IL surface and the binding of the single cations to each other will be higher for **IL3**. Thus, incoming sputtered metal atoms should be impeded more effectively of getting into the IL “bulk” by **IL3** with respect to **IL1**. This means that the nucleation of small metal clusters starts to be intensified on the **IL3** surface with respect to **IL1** since more and more atoms land on the surface. Incoming atoms merge with already-landed atoms/clusters until they are big enough to overcome the IL surface organization and to immerse into the IL “bulk”. So, the IL surface structure already contributes to different NP sizes, which should develop in pure **IL1** and **IL3**, with the bigger NPs being present in **IL3** due to its stronger surface organization. As depicted in Figure 4 and Figure 7b, NPs in **IL3** are bigger [(8.0±3.3) nm] than in **IL1** [(5.4±2.1) nm], supporting these assumptions. After immersion of NPs in the IL “bulk”, another modification of the stabilization effect occurs independent from surface aggregation, since all **IL1/IL3**-mixtures result in bigger maximum NP sizes than pure ILs. It can be assumed that NP stabilization in the IL “bulk” is also influenced by the presence of both IL cations in the mixtures and thus in the outer shell of the stabilizing NP cages. Since [Bmim] has an aromatic character and thus is less coordinative due to its reduced electrostatic strength, it could displace [BmPyr] in the outer cage shell. With the increasing portion of **IL1** in the mixtures and thus the destabilization of the outer shell of the cage, the NP size should increase. This development is not clearly visible in Figure 4 since the mean diameter of the Ag NPs in the mixture 60 vol.% **IL1**/40 vol.% **IL3** is smaller than for the surrounding mixtures 80 vol.% **IL1**/20 vol.% **IL3** and 40 vol.% **IL1**/60 vol.% **IL3**. However, the biggest NP diameters were found in the mixtures with the highest and second highest **IL1** portion. This indicates that above-mentioned effects play a role in **IL1/IL3**-mixtures. Indeed, the smaller mean diameter of NPs in some mixtures contradicts the above-mentioned assumptions, since it would be expected that bigger NPs would also lead to an increased mean diameter. One explanation for this observation is the volume of the NPs. The volume of a sphere increases with its diameter to the power of three, so bigger spheres (or NPs) require more material and thus the remaining material in the ILs could limit the growth of the other NPs to smaller sizes than expected. The fact that NPs in **IL1** are smaller than in **IL3**, although [Bmim] should be less coordinative than the [BmPyr] cation of **IL3**, can be seen as confirmation that the NP formation process on the IL surface is determining the NP size and its further development.

For Au sputtered in **IL1/IL3**-mixtures, the trend for bigger maximum NP sizes is also visible in TEM images, see Figure 5 and Figure 7c. Both IL mixtures with the highest and second highest **IL1** portion show a higher maximum NP size and a bigger mean diameter than all other ILs (mixtures) with Au NPs. However, the stabilization effect of the different surface compositions cannot be identified with the clearness of the Ag NPs. Both pure ILs lead to NPs with the same mean diameter and same standard deviation [(2.2±0.8) nm], but **IL3** shows a higher frequency of NPs with sizes of 3 nm and 5 nm and less NPs with sizes of 1 nm. Thus, the slight difference in mean diameter could be hidden in the rounding of the mean diameter value.

For Cu sputtered in **IL1/IL3**-mixtures, the above-described size development trends for **IL1/IL3**-mixtures could not be fully confirmed, see Figure 6 and Figure 7d. All samples have nearly the same mean NP diameter and comparable size distributions, except the 20 vol.% **IL1**/80 vol.% **IL3** sample. **IL3** and all **IL1/IL3**-mixtures show a tendency to bigger NP diameters with respect to **IL1**. **IL1** has the highest frequency of NPs with diameters of 2 nm and the lowest frequency of NPs with 5 nm and bigger diameters, except for the 80 vol.% **IL1**/20 vol.% **IL3** sample. Here, the frequency of NPs with 3 nm diameters is dominant and the number of NPs with diameters of 4 nm and bigger is comparable to **IL1**. Therefore, it is challenging to clearly identify the trend of **IL1/IL3**-mixtures for enabling bigger maximum NP sizes in the case of Cu NPs, but a weak tendency could be found in the size distribution. In general, the identification of the **IL1/IL3**-mixture effects are challenging for Au and Cu NPs. With respect to Ag NPs in **IL1/IL3**-mixtures, Au and Cu NPs in **IL1/IL3**-mixtures are much smaller in mean diameter and maximum NP sizes and have a narrower size distribution, which hinders identifying a clear trend. Nevertheless, the general trend for bigger maximum NP sizes in the **IL1/IL3**-mixtures with respect to pure **IL1** and **IL3** was detectable for Au and Ag NPs, as depicted in Figure 7.

## 5. Conclusions

A new approach for tuning the stabilization abilities of ILs concerning size distribution of the sputtered NPs was presented. By mixing selected ILs, differing only by either the cation or the anion, two tuning effects have been accomplished: First, the size of the NPs could be influenced in the nanometer regime by mixing two ILs with slightly different anion sizes and thus much finer than using pure but different ILs. Second, the stabilization limits of the pure ILs could be exceeded by mixing two ILs with different cation size and structures due to one cation being based on an imidazolium and the other on a pyrrolidinium ring. Thus, bigger maximum NP sizes could be realized in these IL mixtures. These results show that applying the literature knowledge about the stabilization effect of the different IL ions can be beneficially used for the controlled synthesis of NPs and also confirm the literature knowledge on the formation and stabilization of NPs on and within ILs. Since all mixture samples for the respective element have been synthesized in the same deposition, only the different IL portions are responsible for the observed size effects and thus underline the expressiveness of this new, thus far unconducted NP stabilization process.

## Figures and Tables

**Table 1:** Characteristics of the used chemicals for IL mixtures. Values were provided by the delivering companies, if not noted differently by the addition of the literature sources at the specific values. ^A^ Value estimated by calculating the single particle volume from the total molar volume given by Morgan et al. [62]. ^B^ Value calculated by subtracting known cation volume from total molecule volume. ^C^ Value estimated using density and molar mass of anisole.

Chemical	Purity (%)	Halide Content (ppm)	Water Content (ppm)	Viscosity (mPa s)	Cation Volume (nm^3^)	Anion Volume (nm^3^)	Total Molecule Volume (nm^3^)
**IL1** [Bmim][(Tf)_2_N]	>99	<100	51	49.00 (25 °C)	0.196 ± 0.021 [63]	0.232 ± 0.015 [63]	0.428 ± 0.036 [63]
**IL2** [Bmim][(Pf)_2_N]	>98	<250	60	59.50 (40 °C) 115.90 (25 °C) [64] 153.30 (20 °C) [64]	0.196 ± 0.021 [63]	0.292 ± 0.021 ^B^	0.488 ^A^
**IL3** [BmPyr][(Tf)_2_N]	>99	<100	80	94.00 (20 °C)	0.221 ± 0.015 [63]	0.232 ± 0.015 [63]	0.453 ± 0.030 [63]
Anisole	99	-	<1000	1.52 (15 °C)	-	-	0.181 ^C^

**Table 2:** Sputter deposition parameters. For parameters of Ag deposition in **IL2** and **IL3**, see Meyer et al. [23]. For parameters of Au and Cu deposition in **IL1**, see Meischein et al. [25].

Element in IL Substrate	Start Pressure (Pa)	Ignition Pressure (Pa)	Ignition Power (W)	Pre-Clean Duration (s)	Deposition Pressure (Pa)	Deposition Power (W)	Deposition Duration (min)	Deposition Rate (nm/s)	Film Thickness (nm)
Ag in pure **IL1**	$8.93\times 10^{-5}$	1.33	20	120	0.5	30	40	0.2216	∼530
Ag in **IL1/IL2** mixture	$6.67\times 10^{-5}$	1.33	20	120	0.5	30	40	0.2216	∼530
Ag in **IL1/IL3** mixture	$9.60\times 10^{-5}$	1.33	20	120	0.5	30	40	0.2216	∼530
Au in **IL1/IL3** mixture	$1.11\times 10^{-4}$	1.33	20	120	0.5	30	30	0.2275	∼410
Cu in **IL1/IL3** mixture	$1.13\times 10^{-4}$	1.33	20	240	0.5	30	120	0.080	∼550
